# Real-Time Imaging of Polioviral RNA Translocation across a Membrane

**DOI:** 10.1128/mBio.03695-20

**Published:** 2021-02-23

**Authors:** Krishanthi S. Karunatilaka, David J. Filman, Mike Strauss, Joseph J. Loparo, James M. Hogle

**Affiliations:** a Department of Biological Chemistry and Molecular Pharmacology, Harvard Medical School, Boston, Massachusetts, USA; b Department of Anatomy and Cell Biology, McGill University, Montreal, Quebec, Canada; Columbia University Medical College

**Keywords:** capsid proteins, genome translocation, liposomes, nonenveloped virus, RNA virus, single-particle imaging, fluorescence, poliovirus, virus entry

## Abstract

Genome transfer from a virus into a cell is a critical early step in viral replication. Enveloped viruses achieve the delivery of their genomes into the cytoplasm by merging the viral membrane with the cellular membrane via a conceptually simple mechanism called membrane fusion. In contrast, genome translocation mechanisms in nonenveloped viruses, which lack viral membranes, remain poorly understood. Although cellular assays provide useful information about cell entry and genome release, it is difficult to obtain detailed mechanistic insights due both to the inherent technical difficulties associated with direct visualization of these processes and to the prevalence of nonproductive events in cellular assays performed at a very high multiplicity of infection. To overcome these issues, we developed an *in vitro* single-particle fluorescence assay to characterize genome release from a nonenveloped virus (poliovirus) in real time using a tethered receptor-decorated liposome system. Our results suggest that poliovirus genome release is a complex process that consists of multiple rate-limiting steps. Interestingly, we found that the addition of exogenous wild-type capsid protein VP4, but not mutant VP4, enhanced the efficiency of genome translocation. These results, together with prior structural analysis, suggest that VP4 interacts with RNA directly and forms a protective, membrane-spanning channel during genome translocation. Furthermore, our data indicate that VP4 dynamically interacts with RNA, rather than forming a static tube for RNA translocation. This study provides new insights into poliovirus genome translocation and offers a cell-free assay that can be utilized broadly to investigate genome release processes in other nonenveloped viruses.

## INTRODUCTION

The replication of viruses critically depends on their ability to deliver their genomes into host cells (1). In contrast to virus internalization pathways that mainly rely on the host cell machinery, the mechanisms by which viruses transfer their genomes or nucleoprotein complexes into the cytoplasm through membranes appear to be predominantly virally encoded (2–4). Thus, it is reasonable to hypothesize that genome delivery mechanisms will be conserved among closely related viruses, making these pathways appealing targets for antiviral therapies. Enveloped viruses can transfer their genomes into the cytoplasm by merging their viral envelopes with cellular membranes (5–7). Because nonenveloped viruses lack an external membrane coat, they must instead provide machinery to translocate their genomes across cellular membranes either by forming pores or by disrupting endosomal membranes (8–10). These processes remain poorly understood.

Poliovirus (PV) is an ideal model to study genome translocation in nonenveloped viruses because it is biochemically, genetically and structurally well characterized and it is closely related to a number of other important viral pathogens, including coxsackievirus A16 (CAV16) and enterovirus 71 (EV71) (3). PV, the cause of human poliomyelitis, is a member of the genus *Enterovirus* of the family *Picornaviridae*. The virus consists of a positive-sense single-stranded RNA genome (∼7,500 nucleotides) encapsulated by an icosahedral capsid that contains 60 copies of the four capsid proteins (VP1, VP2, VP3, and VP4) (11, 12). Poliovirus infection is initiated when the virus attaches to its cell surface poliovirus receptor (PVR), CD155 (13). The binding of PV to its receptor at physiological temperature triggers a conformational change of the native virus (160S), which expands to form an altered particle known as the 135S or A particle (14, 15). This conversion results in the externalization of two internal viral peptides: the N-terminal extension of VP1 and the myristoylated capsid protein VP4 (15–17). These externalized peptides insert into cell membranes, allowing virus particles to anchor to the membrane in a receptor-independent manner for subsequent cell entry and genome delivery (17–19). Live-cell fluorescence analysis has shown that the 135S particle is internalized via a noncanonical endocytic pathway, after which the RNA genome is released into the cytoplasm from vesicles close to the plasma membrane (20).

Biochemical and genetic characterizations, together with cryo-electron microscopy (cryo-EM) structures of key poliovirus entry intermediates, provide strong evidence that externalized peptides form membrane-spanning channels to transport the RNA genome into the cytoplasm (18, 21–27). It has been shown that infectivity and the bulk internalized RNA are insensitive to the presence of RNase A in endosomes, suggesting that viral RNA is protected during genome release (28). These discoveries, combined with the observation that empty particles (80S) remain associated with vesicles after genome release (20), strongly support a model in which the virus forms channels or pores in the intact membrane to translocate RNA directly from the interior of the capsid into the cytoplasm.

A thorough understanding of the mechanism of infection requires a detailed characterization of the behavior of individual virus particles that are involved in genome translocation. Previous cellular assays were able to provide insights into the endocytic pathways used for internalization, and the location and average timing of genome release (20). However, those studies were performed using large numbers of virus particles per cell, which was necessitated by the high particle-to-PFU ratio of the viruses and the need to attain sufficient signal in biochemical assays. Unfortunately, such a high multiplicity of infection (MOI) precludes detailed mechanistic analysis due to the prevalence of nonproductive events. To address these issues, we previously developed receptor-decorated liposomes (RDLs) as a simple model system to explore the genome translocation machinery of poliovirus and related viruses (19). These studies showed that poliovirus binds to an RDL (stably at low temperature) and undergoes a conformational rearrangement at physiological temperature that includes externalization of the capsid protein VP4 and the N-terminal region of VP1. Furthermore, it has been shown that these externalized peptides insert into the membrane and promote the release of viral RNA across the membrane, thereby mimicking the early steps of viral infection (19, 22).

Cryo-electron tomographic (cryo-ET) reconstructions of the model virus-membrane complexes, produced by elevating the temperature of virus-receptor-liposome complexes to 37°C, were previously obtained by averaging subtomograms of individual complexes (22). Remarkably, these reconstructions consistently showed one or more direct umbilical connections between a virus particle and a nearby liposome membrane. Notably, these altered virus particles had variable amounts of RNA inside them, and several of the complexes showed visual evidence for viral RNA translocation across the membrane and into the lumen of the liposome (22, 28). Collectively, these data suggest that viral RNA unfolds at least transiently, transfers across the capsid and the membrane as single-stranded RNA, within a protective structure, and then refolds after the translocation (20, 29).

To probe the mechanism of RNA translocation across a membrane, we have developed a liposome-based fluorescence assay by capturing YoPro-1 (a nucleic acid-binding dye whose fluorescence increases upon binding RNA) in the lumens of RDLs (28). Our initial experiments showed that, with poliovirus added, the integrated fluorescence intensity increases over time when temperature ramps from room temperature (RT) to 42°C. Furthermore, it was demonstrated that viral RNA translocation is insensitive to RNase A (28). Although that study was able to provide evidence for RNA translocation across a membrane, the use of untethered liposomes at a relatively high virus-to-liposome ratio made it difficult to track individual translocation events.

In the present study, we characterized the mechanism of viral genome translocation across a membrane by monitoring the genome release of individual viruses in real time using surface-tethered RDLs that had bound exactly one virus per liposome. These single-particle imaging results suggest that genome release is a complex process that may include multiple rate-limiting steps. We found that the efficiency of genome translocation across the model membrane was comparatively low at the single-particle level *in vitro*. However, the addition of exogenous N-terminally myristoylated VP4 enhanced the efficiency and extent of this process, which further supports the idea that VP4 plays a vital role in genome translocation. Together, the biochemical and structural results suggest that VP4 promotes genome translocation by interacting with RNA to form a protective connection between the virus surface and the membrane, forming a membrane-spanning channel, and perhaps by facilitating the unfolding of the secondary structure of the RNA. The ability of exogenously added VP4 to facilitate RNA translocation together with the high particle-to-PFU ratio of poliovirus and other enteroviruses further suggests that VP4 from multiple virions may participate in productive RNA release and raises the exciting possibility that this process may be dynamic, with VP4 repeatedly attaching to, releasing, and reattaching to the viral RNA in transit, rather than forming a static tube that the RNA slides through. Finally, we believe that our newly developed ability to obtain that kind of mechanistic detail, using a tethered-liposome model, represents a valuable technological advance that will be broadly applicable to other nonenveloped viruses.

## RESULTS

### RDLs provide a cell-free model to study genome translocation.

RDLs were constructed using a mixture of phospholipids and cholesterol to mimic the biological membranes in cells. The ectodomain of the poliovirus receptor (PVR) containing a C-terminal six-histidine tag was anchored to the surface of the liposome through a small fraction of lipids containing nickel-nitrilotriacetic acid (Ni-NTA) head groups as previously described (19). To detect the virus genome translocation across a membrane in real time, RDLs were prepared in the presence of TOTO-3, a nucleic acid binding dye, to trap the dye inside the lumen of the liposomes. In addition to our lipid mixture, a small fraction of polyethylene glycol (PEG)-biotin-labeled lipids was added to the membrane to immobilize RDLs on a streptavidin-coated slide for time-lapse imaging.

**FIG 1:**
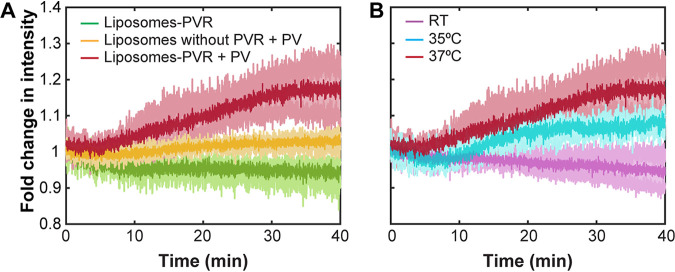
PV efficiently translocates genomic RNA across synthetic vesicle membranes in a temperature-dependent manner. (A) Bulk fluorescence measurements of the fold change in fluorescence intensity of TOTO-3 containing receptor-decorated liposomes (liposomes-PVR) with time in the absence of poliovirus (green) and in the presence of poliovirus at 37°C (red). The yellow line represents the fold change in intensity of liposomes without PVR in the presence of poliovirus at 37°C. (B) Effect of temperature on viral RNA translocation. Bulk fluorescence intensities were measured at RT (purple), 35°C (cyan), and 37°C (red; from panel A). Data are averages and standard deviations (shaded regions) from 2 or 3 independent experiments.

We confirmed that PV is able to inject its genome into these RDLs using a bulk fluorescence assay (Fig. 1). The change in fluorescence intensity was normalized based on the initial intensity of RDLs and reported as fold change in intensity over time. In the absence of poliovirus, RDLs alone showed a slight decrease in the fluorescence intensity over time compared to the initial intensity (Fig. 1A, green), presumably due to photobleaching. In contrast, the fluorescence intensity of the TOTO-3 within the RDLs increased ∼1.2-fold with time in the presence of poliovirus at 37°C, and this increase was dependent on PVR (Fig. 1A, red and yellow). Previous reports found that PV genome release depended on temperature (19, 20, 28). We sought to investigate the effect of temperature on viral RNA release using our RDL system. Consistent with previous findings, genome translocation appeared to be quite sensitive to temperature with our assay. At room temperature (RT), TOTO-3-containing RDLs showed a slight decrease in intensity in the presence of virus, which was similar to that of liposomes alone (Fig. 1B, purple). Interestingly, a 2°C difference in temperature made a substantial difference in the degree of genome translocation. The fold change in intensity at 35°C was about half of the change seen at 37°C, and the initiation of translocation was slightly delayed at 35°C by a few minutes (Fig. 1B, red and cyan). All of these bulk fluorescence assays were performed with RNase A present in the solution, outside RDLs, as previously described (28). This was done to confirm that the observed increase in fluorescence intensity of samples was caused by the enhancement of TOTO-3 signal due to the binding of viral RNA that was translocated across the vesicle membranes.

### Imaging of RNA translocation at one virus per liposome.

**FIG 2:**
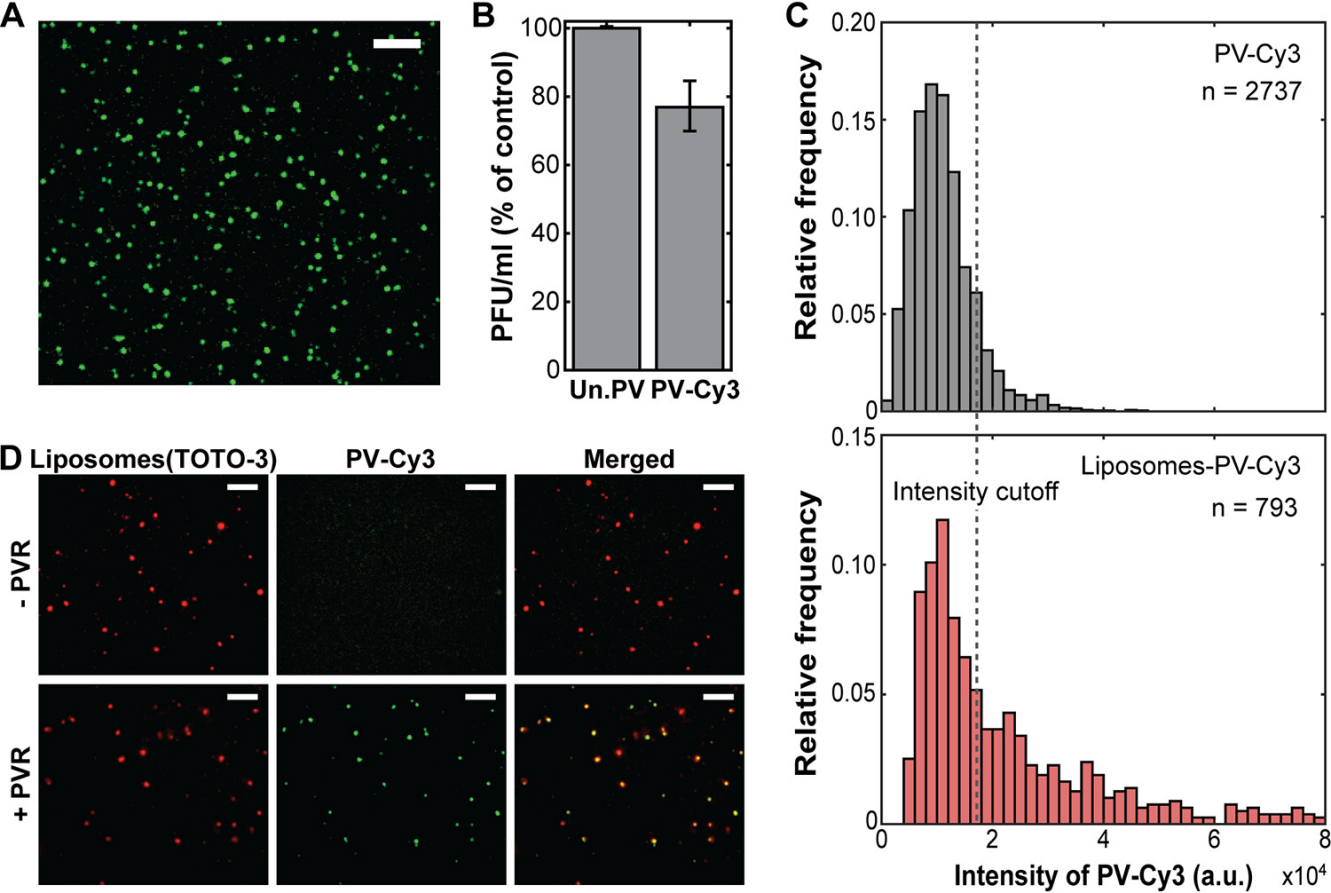
Cy3-labeled poliovirus specifically binds to the surface-tethered receptor-decorated liposomes. (A) Cy3-labeled poliovirus. Bar, 5 μm. (B) Infectivity of unlabeled and Cy3-labeled poliovirus in cells. (C) Intensity distribution of individual multilabeled poliovirus particles with Cy3 (gray [top]) and intensity distribution of Cy3 at individual liposome-poliovirus complexes (red [bottom]), indicating the number of poliovirus bound per single liposome. The dashed line indicates the intensity cutoff used to identify the liposomes containing one virion. (D) Fluorescence images of the specific binding of PV-Cy3 (green [middle]) on TOTO-3-containing liposomes (red [left]) with (top) and without (bottom) PVR and merged images of both channels (right). Bars, 5 μm.

To reveal the mechanism of viral genome translocation across a membrane, it is essential to monitor the genome translocation process in real time using one virus per liposome. As the first step to achieve this goal, purified poliovirus was fluorescently labeled with amine-reactive Cy3 fluorophore (Fig. 2A) to visualize the virus particles (20). Virus particles were multiply labeled with Cy3 (∼20 Cy3 dye molecules per PV) to visualize 100% of the particles without significantly affecting the infectivity (Fig. 2B). In order to determine the binding of one virus per liposome, first we monitored the intensity distribution of multilabeled individual virus particles that were nonspecifically bound to a slide surface (Fig. 2C, top). An intensity cutoff value for a single virus particle was experimentally determined based on the observed average intensity and standard deviation, and this intensity cutoff (dashed line) was used to identify liposomes with one attached virus particle (Fig. 2C, bottom). Then, the amount of Cy3-labeled poliovirus (PV-Cy3) required per reaction was optimized to obtain at least ∼50% of virus bound liposomes having one virus particle on the surface.

**FIG 3:**
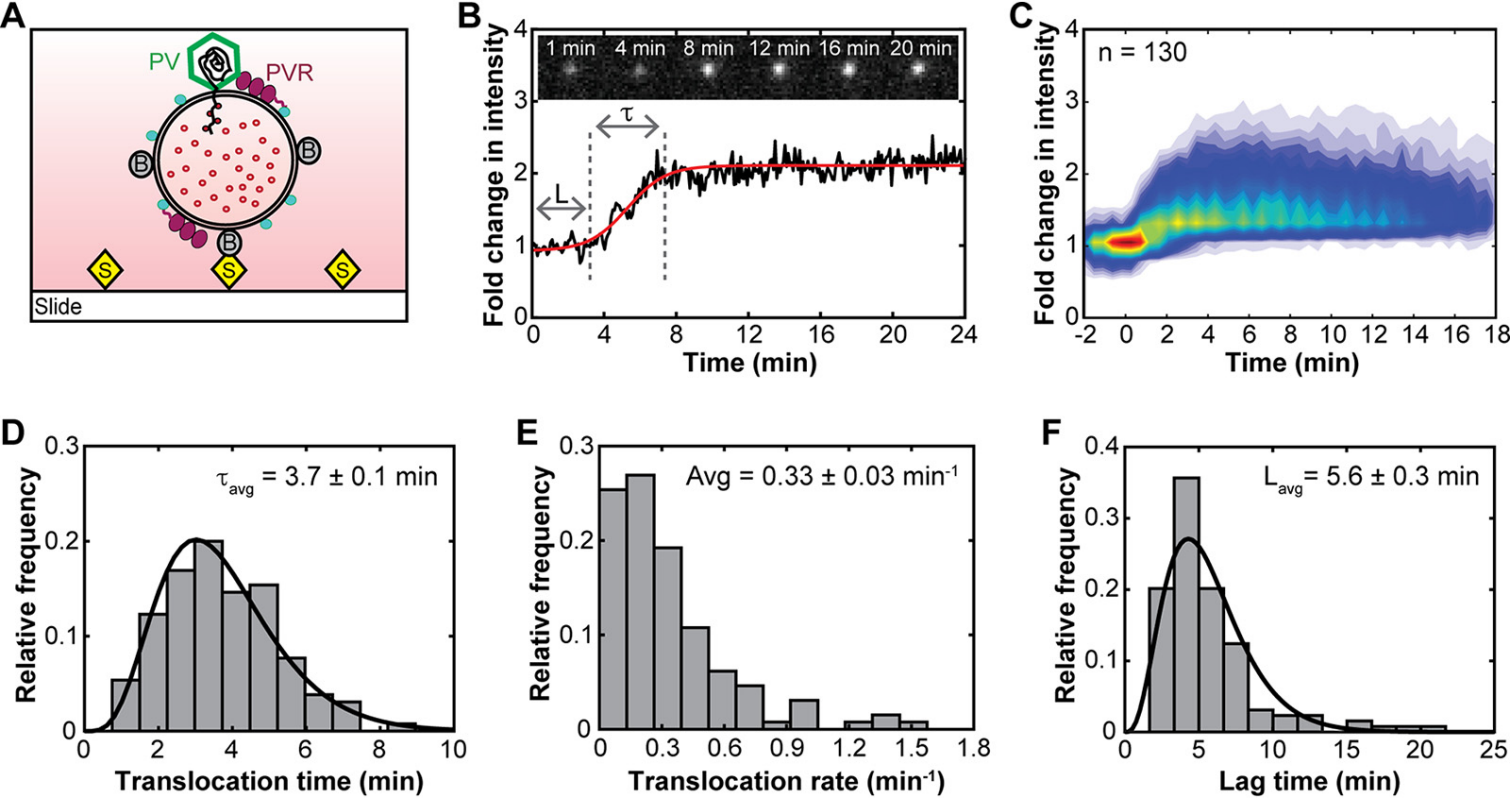
Viral genome translocation at the single-particle level. (A) Experimental setup of a biotinylated receptor-decorated liposome-poliovirus complex surface immobilized on a streptavidin-coated slide for single-particle imaging. The liposome contains the nucleic acid-binding dye TOTO-3 for real-time observation of RNA translocation across the membrane. (B) A typical fluorescence trajectory depicting the fold change in TOTO-3 fluorescence intensity as a single PV translocates its RNA into the vesicle. L and τ represent the lag time and the translocation time, respectively. The red line is a sigmoidal fit to the trajectory. Fluorescence images at the top show the corresponding enhancement of TOTO-3 intensity at indicated times. (C and D) Heat map of synchronized RNA translocation events (C) and corresponding distribution of translocation times observed with PV-RDL alone (D). The black line is the gamma fit. (E) Frequency distribution of translocation rates from individual viruses. (F) Distribution of lag times between incubation starting point and translocation initiation from individual particles. The black line represents a gamma fit. Images were constructed using data observed from PV-RDL complexes at 37°C.

In order to monitor individual viral RNA translocation events in real time using time-lapse imaging, TOTO-3-containing RDLs were immobilized on a streptavidin-coated slide via biotin-streptavidin linkages (Fig. 3A). After washing away unbound liposomes and untrapped TOTO-3 molecules, PV-Cy3 was added to the surface-tethered RDLs to form PV-RDL complexes. To confirm that fluorescently labeled virus particles specifically interact with the membrane via its natural receptor, PVR, the binding of PV-Cy3 on the membrane was monitored using liposomes decorated with and without soluble PVR (Fig. 2D). In the absence of PVR, we did not observe interactions between liposomes and PV-Cy3 (Fig. 2D, top). However, fluorescent signals from both PV-Cy3- and TOTO-3-containing vesicles were observed using vesicles that were decorated with PVR (Fig. 2D, bottom), which indicates that PV-Cy3 specifically interacts with the receptor on the membrane. Fluorescently labeled liposomes with a small fraction of lipids containing rhodamine were used to confirm that TOTO-3 was successfully trapped inside the RDLs (see Fig. S1A in the supplemental material). The fluorescence intensity of TOTO-3-containing PV-RDL complexes was measured over time at 37°C after removal of unbound virus particles (Fig. 3). To probe the mechanism of viral genome translocation across a membrane, we excluded liposomes with no virions attached and the small fraction of liposomes with multiple virions attached and analyzed the intensity enhancement only of PV-RDLs containing one virion per liposome. Figure 3B shows a typical intensity trajectory obtained using single-particle imaging of a single PV-RDL complex at physiological temperature. Upon heating the PV-RDL at 37°C, the fold change in intensity increased over time after a short lag time (L), due to the enhancement of TOTO-3 fluorescence upon binding to translocated viral RNA inside the liposome. The translocation time (τ), corresponding to the time required for a virus to translocate its genomic RNA across the membrane and into the lumen of a vesicle, was obtained from each individual single-particle intensity trajectory, as explained in Materials and Methods.

10.1128/mBio.03695-20.1 FIG S1 Receptor-decorated liposomes entrap TOTO-3 for single-particle imaging of RNA translocation. (A) Fluorescence images of TOTO-3 (red, middle) containing liposomes labeled with a small fraction of lipids containing rhodamine (green, left) and merged image (right). Bars, 5 μm. (B) Average fold change in intensity of TOTO-3 containing RDLs observed with time in the absence of poliovirus at 37°C. Shading indicates the standard deviations of the data points. (C) Distribution of fold change in intensity of liposomes observed after 10 min. The error bar indicates the standard deviation. Download FIG S1, JPG file, 0.2 MB. Copyright © 2021 Karunatilaka et al. 2021 Karunatilaka et al. https://creativecommons.org/licenses/by/4.0/ This content is distributed under the terms of the Creative Commons Attribution 4.0 International license.

To assess the viral genome translocation in a population of virus particles, intensity trajectories obtained from 130 PV-RDL complexes, each containing one PV per liposome, were synchronized as shown in the heat map of Fig. 3C. Consistent with bulk fluorescence results, RDLs alone (in the absence of virus) did not cause an increase in the TOTO-3 intensity at 37°C (Fig. S1B and C). A small reduction in intensity resulted over time due to the photobleaching of dye molecules inside liposomes during fluorescence imaging. To minimize the effect of TOTO-3 photobleaching, all single-particle imaging experiments were performed in the presence of an oxygen-scavenging system. Although we did not observe a significant amount of untrapped TOTO-3 outside the liposomes after washing the sample channel (Fig. S1A), RNase A was added to the solution outside the liposomes to minimize any background fluorescence due to binding of TOTO-3 with RNA contaminants.

### Polioviral genome translocation is a complex multistep process.

Mechanistic insights into genome release can be obtained by analyzing the genome translocation events that are measured from individual virus particles. Analysis of 130 translocation events showed a broad distribution of translocation times, with an average of 3.7 ± 0.1 min at 37°C (Fig. 3D). To extract kinetic information about genome translocation, we measured the RNA translocation rates for individual virus particles by linear fitting the slopes of their time trajectories (e.g., the intensity slope within τ in Fig. 3B) and reported these slopes as the change in normalized fluorescence intensity per minute (Fig. 3E). The majority of poliovirus particles exhibited an overall RNA translocation rate that was low, with an average rate of 0.33 ± 0.03 min^−1^ at 37°C.

To further characterize the genome translocation process, we measured the lag times that preceded the observed genome translocation (Fig. 3B, L). If viral genome release was a simple process consisting of a single rate-limiting step per virion, we would expect to see an exponential distribution of lag times (30). Instead, the histogram of the observed lag times shows a rise followed by a decay, suggesting the existence of multiple steps occurring between virus-receptor binding and the initiation of RNA translocation. Analysis of 130 PV-RDLs showed an average lag time of 5.6 ± 0.3 min (Fig. 3F). The distribution of lag times together with the observed translocation times and translocation rates suggests that viral genome release is a complex process that likely consists of multiple steps.

### Exogenous VP4 enhances the efficiency of genome translocation.

Surprisingly, the efficiency of viral genome translocation was very low when only one virion was present. Indeed, only ∼3 to 4% of observed RDLs with one PV showed an enhancement of fluorescence intensity, indicating the presence of translocated RNA inside the liposomes. In contrast, in our previous report, PV-RDLs containing multiple viruses per liposome showed a significant intensity enhancement in the majority of PV-RDL complexes (28). Furthermore, previous poliovirus studies performed in live cells at low MOI (1 PFU/cell, corresponding to ∼300 to 400 poliovirus particles bound per cell) indicated that RNA release is an efficient process *in vivo* (20). In addition to the possibility of a host cell factor(s) that can enhance the RNA release in cells, these results collectively suggest that some soluble factor(s) available in the presence of multiple viruses might play a role in enhancing the efficiency of RNA release. It has been shown that formation of the 135S-like particles, and therefore the release of the majority of capsid protein VP4, occurs at the cell surface before virus internalization and RNA release (20). Given that there are hundreds of virus particles at the cell surface during infection even at low MOI, it is reasonable to assume that VP4 proteins are abundant in the cell membrane prior to endosome formation and virus internalization. Therefore, the N-terminally myristoylated PV capsid protein VP4 provides an attractive candidate to contribute to efficient RNA translocation, since it becomes externalized during viral infection, binds to membranes (18, 19), and has been shown to play a role in the release of viral RNA across membranes during infection (18). To test this possibility, we observed RNA translocation at the single-particle level in the presence of exogenously added synthetic VP4. In addition, the VP4 concentration dependence of the kinetic measurements *in vitro* serves as a crucial probe of the RNA transfer mechanism.

**(i) Wild-type VP4-N45.** Since the full-length VP4 was poorly soluble, we tested the effect of adding exogenous VP4 on RNA translocation using a myristoylated synthetic peptide that corresponded to the N-terminal 45 amino acids of VP4 (VP4-N45). The first 45 amino acids of VP4 form an intricate stable-looking pentameric complex on the inner surface of mature 160S poliovirus and other related viruses, including rhinoviruses (11). Furthermore, it has been shown that both the full-length VP4 and the N-terminal 45-mer of VP4 from rhinovirus can induce size-selective pores in liposomes, but not the C-terminal 45-mer, and that the presence of the N-terminal myristoyl modification improves this activity (31).

**FIG 4:**
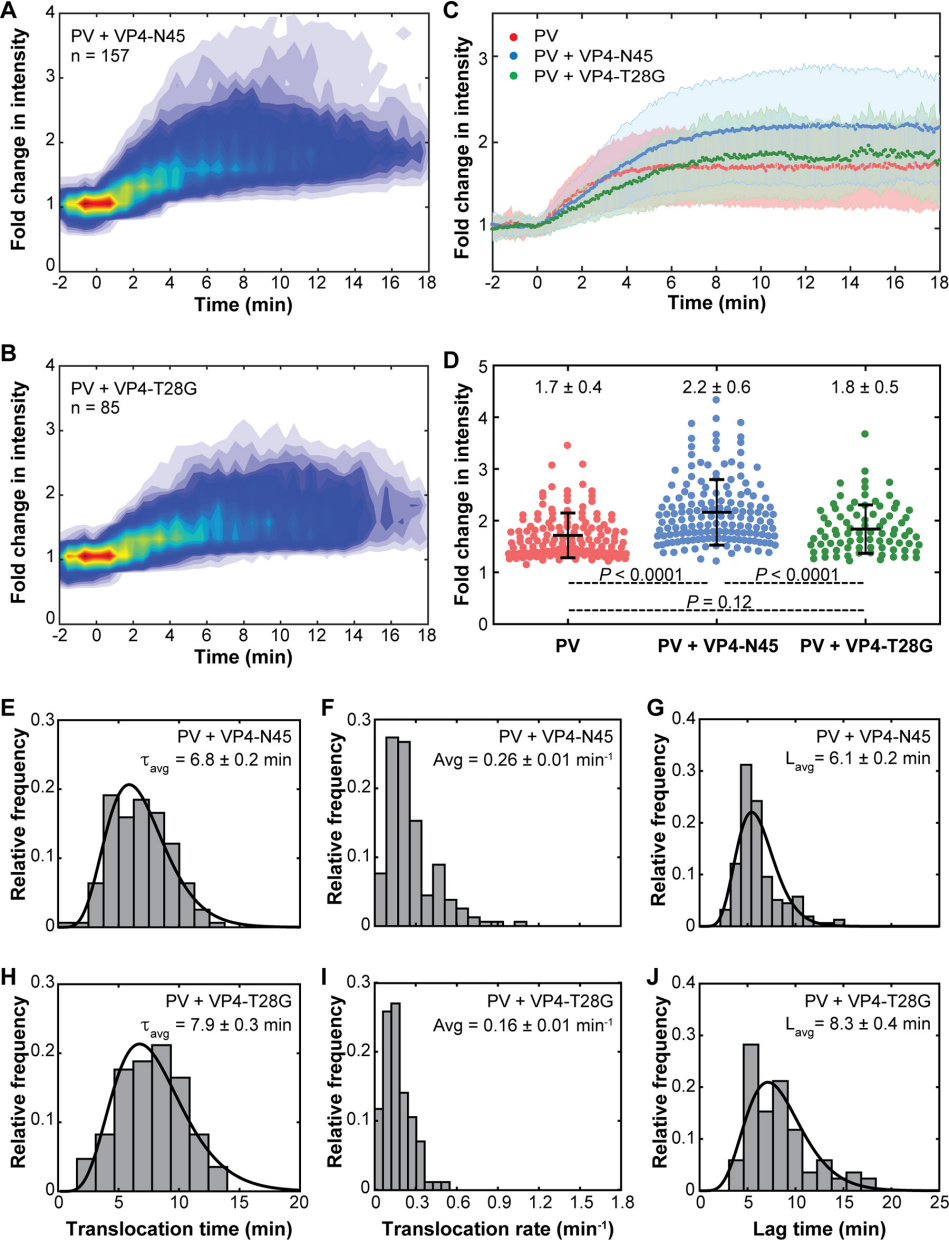
Exogenous wild-type VP4 increases the efficiency of viral genome translocation. (A and B) Heat maps of synchronized RNA translocation events observed for PV with wild-type VP4-N45 and mutant VP4-T28G as indicated. (C) Comparison of the observed average fold change in intensity of PV-RDL complexes with and without exogenous wild-type and mutant VP4 45-mer at 37°C. Shading indicates the standard deviation of the data points. (D) Distribution of maximum fold change in intensity of PV-RDL complexes observed after 10 min of viral RNA translocation. Error bars indicate standard deviations. (E and H) Distributions of translocation times observed in PV-RDL with wild-type and mutant exogenous VP4 45-mer, respectively. Black lines represent the gamma fit. (F and I) Distributions of individual translocation rates indicating the fold change in intensity per minute. (G and J) Distributions of lag times with corresponding gamma fits (black) indicated.

The addition of exogenous wild-type VP4-N45 significantly increased the efficiency of RNA release, with 16% of the liposomes that had one copy of PV undergoing an increase of fluorescence intensity over time in the presence of added VP4, versus 3 to 4% in the absence of added VP4. Figure 4A displays the heat map constructed by computationally synchronizing the translocation starting points of 157 individual intensity trajectories obtained in the presence of VP4-N45 (example trajectories are shown in Fig. S2). Comparison of the observed average fold change in fluorescence intensity of PV-RDLs in the presence and absence of VP4-N45 clearly implies that exogenous VP4 promotes the viral RNA translocation and allows more RNA delivery into the liposomes (Fig. 4C, red and blue). An analysis of maximum fold change in intensity of PV-RDLs observed after 10 min further supports the role of VP4, as it shows average fold changes in intensity of 1.7 ± 0.4 and 2.2 ± 0.6 in the absence and presence of wild-type VP4-N45, respectively (Fig. 4D, red and blue). As observed for liposomes alone, RDLs with exogenous VP4, but without PV, did not increase the fold change in TOTO-3 intensity at 37°C (Fig. S3).

10.1128/mBio.03695-20.2 FIG S2 Single-particle imaging of PV-RDL complexes with and without exogenous VP4. Typical single-particle fluorescence trajectories depicting increases in fluorescence intensity as individual virus particles translocate their genomes across the vesicle membrane. Fluorescence intensity changes were recorded in the (A) absence or (B) presence of exogenous wild-type VP4-N45 peptide under physiological conditions. Download FIG S2, JPG file, 0.4 MB. Copyright © 2021 Karunatilaka et al. 2021 Karunatilaka et al. https://creativecommons.org/licenses/by/4.0/ This content is distributed under the terms of the Creative Commons Attribution 4.0 International license.

10.1128/mBio.03695-20.3 FIG S3 VP4 did not significantly enhance the fluorescence intensity of liposomes in the absence of poliovirus. (A) Average fold change in intensity of TOTO-3-containing receptor-decorated liposomes with time in the presence of VP4-N45 without poliovirus at 37°C (grey, *n* = 200). Shading indicates the standard deviations of the data points. (B) Distribution of maximum fold change in intensities of individual liposomes observed after 10 min in the presence of exogenous VP4-N45 with (blue; Fig. 4D) and without (grey) poliovirus at 37°C. Error bars indicate standard deviations. Download FIG S3, JPG file, 0.2 MB. Copyright © 2021 Karunatilaka et al. 2021 Karunatilaka et al. https://creativecommons.org/licenses/by/4.0/ This content is distributed under the terms of the Creative Commons Attribution 4.0 International license.

Not only did exogenous VP4-N45 increase the amount of RNA delivered into the liposome, it also increased the translocation time. Specifically, the addition of wild-type VP4 45-mer peptide to PV-RDLs increased the average translocation time from 3.7 ± 0.1 min to 6.8 ± 0.2 min (compare Fig. 3D and Fig. 4E; *P < *0.0001). Note, however (Fig. 4C, red and blue curves), that the slopes of the curves with and without wild-type VP4 are very similar to one another, suggesting similar translocation times per nucleotide. Thus, the apparent increase in translocation time may be due entirely to the increase in the amount of RNA translocated, at a constant rate. Unwinding of RNA at a low rate, to form single-strand RNA (ssRNA) prior to traversing the capsid, was previously proposed (32) to explain the distribution of densities inside populations of 80S particles. To further understand the effect of VP4 on RNA release kinetics, RNA translocation rates were obtained for all individual events observed with wild-type VP4-N45 (Fig. 4F). On average, PV in the presence of exogenous wild-type VP4-N45 exhibited an overall RNA translocation rate of 0.26 ± 0.01 min^−1^ (Fig. 4F), which is slightly lower than that observed with PV in the absence of added VP4 (0.33 ± 0.03 min^−1^) (Fig. 3E). The distribution of lag times in the presence of exogenous wild-type VP4 45-mer also showed a rise-and-decay pattern similar to that seen using PV-RDL alone (Fig. 4G). Interestingly, the addition of wild-type VP4-N45 peptide did not significantly affect lag times, as the average of 6.1 ± 0.2 min was similar to the lag times for PV in the absence of exogenous peptide (Fig. 3F and Fig. 4G; *P = *0.133).

**(ii) T28G mutant VP4-N45.** To test the specificity of the observed effect of VP4 on viral RNA translocation, we characterized RNA release in the presence of the T28G mutant VP4 N-terminal 45-mer (VP4-T28G), which substitutes glycine for threonine-28 of VP4. Previous studies have shown that while the T28G mutant is lethal, its virions can be produced by transfection with viral RNA that contains the mutation. The mutant virions bind to and are internalized into cells, but the internalized virions fail to release their genomes into the cytoplasm (18). In agreement with these findings, our single-particle experiments performed in the presence of VP4-T28G show that the mutant peptide does not improve the efficiency of RNA translocation. Thus, exogenous VP4-T28G showed enhanced fluorescence in 6 to 7% of the liposomes versus 16% with wild-type peptide and 3 to 4% in the absence of exogenous peptide.

Comparison of heat maps constructed by synchronizing individual intensity trajectories in the presence of wild-type and mutant VP4 45-mer clearly indicate that mutant VP4 did not enhance RNA translocation in a manner similar to that of wild-type peptide (Fig. 4A and B). Moreover, the mutant VP4-T28G did not enhance translocation beyond what was observed using PV-RDLs alone. Similarity to the VP4-free control can be seen both in the average fold change in intensity traces and in the maximum fold change in intensity distributions (Fig. 4C and D, red and green). In contrast, wild-type VP4-N45 significantly increased the fluorescence intensities of RDLs over time at a single-particle level (Fig. 4C and D, blue). The failure of the mutant VP4 45-mer to improve the extent of RNA translocation rules out the possibility that the enhancement seen with wild-type 45-mer was due to nonspecific effects such as the presence of the myristoyl group or to the presence of lysines that were added to the peptide to increase its solubility. This specificity strongly argues that the effects seen using wild-type peptide are biologically relevant.

The addition of T28G mutant VP4 peptide significantly increased the observed average translocation time to 7.9 ± 0.3 min (Fig. 4H), compared to that of PV alone (3.7 ± 0.1 min; *P < *0.0001) or with exogenous wild-type VP4 peptide added (6.8 ± 0.2 min; *P < *0.0001). Although PV-RDLs in the presence of exogenous VP4-T28G showed final intensities that were similar to those seen using PV-RDL alone, mutant VP4-T28G had a distinctly negative effect on the rate and extent of intensity increase (Fig. 4C). In order to understand the effect of T28G mutant VP4 on RNA release kinetics, RNA translocation rates were compiled for all individual translocation events observed using mutant peptide (Fig. 4I). Compared to PV alone or to PV in the presence of exogenous wild-type VP4, the addition of mutant VP4-T28G consistently decreased the RNA translocation rates, showing a narrowed distribution with an average translocation rate of 0.16 ± 0.01 min^−1^ (Fig. 4I).

Consistent with the distribution of lag times that were observed using PV-RDL either alone or with added wild-type VP4-N45, the addition of mutant peptide produced a similar nonexponential distribution, showing a rise and decay pattern (Fig. 3F and 4G and J). However, the addition of mutant VP4-T28G to PV-RDL (which already contained endogenous wild-type VP4) clearly affected the observed lag times, increasing the average lag time to 8.3 ± 0.4 min (Fig. 4J), relative to lag times using PV alone or PV with wild-type VP4-N45. Although the added wild-type VP4 did not significantly affect RNA release kinetics, mutant VP4-T28G hindered both lag times and translocation rates, which suggests a possible dominant negative effect.

### The majority of viruses show an incomplete genome translocation *in vitro*.

**FIG 5:**
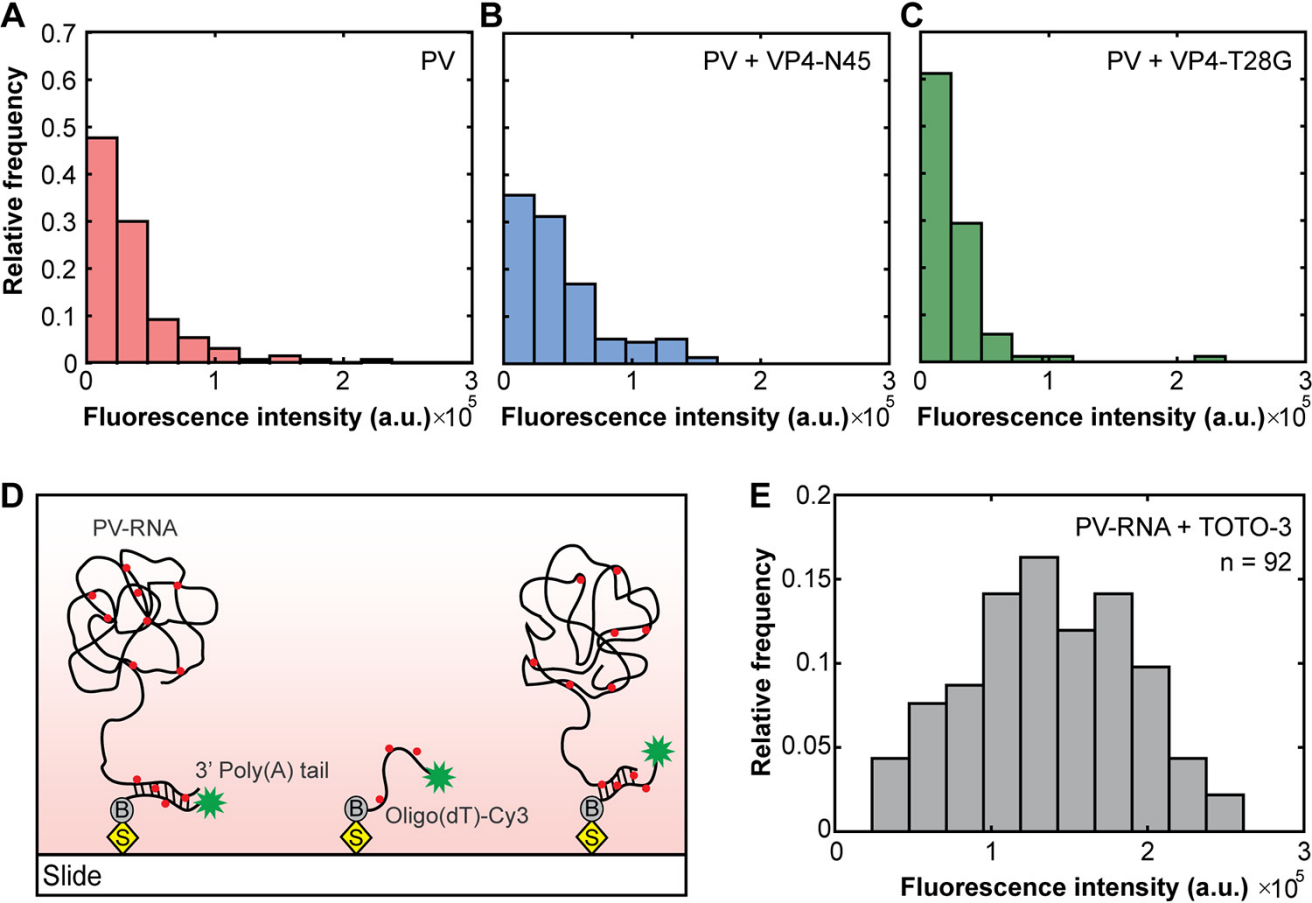
The majority of virus particles show an incomplete genome translocation *in vitro*. (A to C) The distribution of absolute TOTO-3 intensity enhancement of PV-RDL complexes after 10 min without exogenous VP4 (red), with wild-type VP4-N45 (blue), and with mutant VP4-T28G (green). (D) Purified polioviral RNA was surface immobilized on a streptavidin-coated slide by hybridizing with a biotinylated and Cy3-labeled oligo(dT)20 strand for single-molecule imaging of TOTO-3 binding. (E) Fluorescence intensity distribution of individual PV-RNA-TOTO-3 complexes.

The addition of VP4-N45 enhanced the fluorescence intensity of some PV-RDLs, indicating that exogenous wild-type VP4 promotes the translocation of more RNA into liposomes than PV alone. Since we were imaging the genome release using one virus per liposome, the increased fluorescence suggests the possibility of incomplete genome release for some of the viruses. To compare the efficiency of genome release among viruses under different conditions, we subtracted the initial fluorescence intensity from the maximum fluorescence intensity that was observed after 10 min to obtain the intensity enhancement that results from dye binding to translocated RNA (Fig. 5A to C). The intensity distribution using PV-RDL alone showed that the majority of complexes had a low intensity enhancement overall, suggesting a partial genome release (Fig. 5A). As expected, the addition of wild-type VP4-N45 peptide increased the overall fluorescence intensity of liposomes relative to PV-RDL alone. Nevertheless, we observed that the majority of liposomes still exhibited lower intensity enhancement, while only a small population of complexes showed higher intensity enhancement (Fig. 5B). In contrast, the addition of mutant VP4-T28G showed enhancement similar to that of PV alone (Fig. 5C), indicating that the T28G mutation is incapable of promoting additional translocation of RNA into the vesicles.

To further investigate the relationship between the observed fluorescence intensity enhancement and the relative amount of genome release among viruses, we investigated the binding of TOTO-3 directly to purified poliovirus RNA. As shown in Fig. 5D, purified PV-RNA (∼7,500 nucleotides [nt]) was immobilized on a streptavidin-coated slide by hybridizing it with a 20-nt biotinylated oligo(dT)-Cy3 strand complementary to the 3′ poly(A) tail of viral RNA. After addition of TOTO-3 dye, the intensities of PV-RNA-TOTO-3 complexes were measured as described in the methods (Fig. 5E). The peak fluorescence of the enhanced intensity distribution of PV-RDLs alone or in the presence of added wild-type or mutant peptide overlapped at the lower end of the distribution of fluorescent intensities observed for tethered free PV-RNA, further supporting that the translocation is incomplete in the majority of cases but clearly enhanced with the addition of wild-type VP4 peptide (Fig. 5).

## DISCUSSION

Although understanding the mechanism of viral genome translocation has great potential to identify targets for therapeutic development, the mechanisms by which nonenveloped viruses translocate their genomes through a membrane into the host cytoplasm remain poorly characterized. Despite the existence of several alternative models, a number of lines of evidence suggest that poliovirus and closely related members of the enterovirus genus translocate their genomes through intact membranes via channels formed by membrane-associated capsid proteins (18, 22, 28, 33, 34). However, in the past, it was difficult to fully capture the mechanism of viral genome translocation because it was typically studied indirectly through bulk infectivity assays or through electron microscopy snapshots, which provide key structural information but lack kinetic insights. To reveal the exact mechanism of viral genome translocation across a membrane, it is necessary to monitor genome release by individual viruses in real time. Technical difficulties and sensitivity of the viral genome to external manipulations make the direct visualization of genome release *in vivo* challenging (20). For that reason, we developed a cell-free, TOTO-3-containing receptor-decorated liposome model to investigate the translocation of a viral genome across a membrane at the single-particle level.

Real-time quantification of TOTO-3 intensity enhancement, corresponding to the binding of translocated RNA inside the vesicles, not only provided evidence for genome transfer through an intact membrane but also allowed us to acquire mechanistic information. Comparison of bulk fluorescence assays that were performed using PV and liposomes with and without PV receptor demonstrated that initial receptor-mediated binding of PV is required for the subsequent efficient transfer of the viral genome through a membrane at physiological temperature. Consistent with previous studies of temperature-triggered PV conversion in the presence of RDLs (19), an evaluation of PV-RDL fluorescence intensities at different temperatures indicates that genome release is a temperature-dependent process. Thus, although PV can bind to PVR on the membrane at room temperature, higher temperatures, closer to the physiological case, are essential to initiate subsequent steps of genome release.

Viral genome release is a process that is virtually impossible to synchronize physically, and thus, release kinetics are obscured when many viruses deliver their genomes into cells or vesicles at the same time. To overcome this, we monitored the intensity enhancement of surface-tethered PV-RDLs in real time using one PV per liposome at 37°C. Analysis of individual virus-attached vesicles shows that PV transfers its genome through the membrane and into the lumen of an RDL after a short lag time. As expected, the rise-and-decay shapes of the lag time distributions indicate the existence of multiple rate-limiting steps associated with the initiation of RNA release. Previous studies have shown that ∼3 min is sufficient for complete conversion of a native virus population to 135S particles when they are incubated in the presence of receptor at 37°C (35). Therefore, the observed average lag time of ∼6 min suggests the presence of additional steps subsequent to the formation of 135S particles. The obvious candidate for the requirement for additional time is the symmetry-breaking formation of an umbilical connection that presumably includes RNA and protein, which connects the interior of the virus with the interior of the liposome (22). These structures were previously observed when PVR-membrane complexes were heated to 37°C, and they have been postulated to be involved in the RNase-protected transfer of viral RNA through the endosomal membrane (28).

The finding that RNA translocation is a complex process with multiple rate-limiting steps is not entirely surprising. Previously, the dissociation of Syto82 intercalating dye from PV-RNA during genome release in live cells suggested that the disruption of RNA secondary structure is required for RNA release (20). Hence, we propose that genome translocation consists of an initiation burst followed by propagation that involves a series of RNA unwinding and translocation steps. Although our cell-free system was capable of capturing translocation of the viral genome in real time, it is important to note that the majority of PV virions may not transfer their complete genomes into the vesicles *in vitro*. Incomplete translocation of the genome could be due to a number of possible reasons, including the curvature of the membrane and the small size of the liposomes. In addition, incomplete release may also occur because additional cellular factors are needed that facilitate complete genome translocation during infection. Indeed, it was recently shown that host factors, such as the lipid-modifying enzyme PLA2G16, are required to facilitate viral genome translocation in live cells (36).

Despite the fact that PV-RNA release is highly efficient in live cells (20), and in previous bulk fluorescence studies with multiple virions per liposome (28), in the present study, only ∼3 to 4% of observed RDLs containing one PV showed an intensity enhancement that corresponded to RNA translocation at 37°C. The addition of exogenous wild-type VP4-N45 not only increased the number of detectable RNA release events by ∼4-fold but also increased the amount of translocated RNA seen inside the liposomes at the single-particle level. Interestingly, a single mutation in the VP4, T28G, which has been shown to prevent RNA release, did not similarly enhance the efficiency of RNA translocation. Overall, these results provide evidence that VP4 plays a specific role during genome release and can function in *trans*. However, the exact mechanism by which VP4 promotes RNA transfer across a membrane (and likely serves as an essential buffer between membrane lipids and RNA charges) needs further investigation.

Besides the proposed channel-forming capability of PV VP4 (18, 37, 38), purified VP4 has been shown to form fibrous aggregates *in vitro* (38) (Fig. S4A and B). Additionally, heat treatment of virions at elevated temperature (50 to 55°C) to induce RNA release results in the formation of very long RNase-resistant fibers that contain both protein and RNA (39, 40) (Fig. S4C and D), including large amounts of VP4 (Fig. S4E). Unfortunately, the fibers formed either by VP4 peptides alone or by heating virions lack long-range periodicity, which makes structural analysis very difficult. However, preliminary cryo-EM studies of both types of filaments are consistent with short-range repeats whose dimensions are strongly suggestive of pentamers of VP4 (Fig. S4B and F). Based on cryo-ET studies of virus-RDL complexes warmed to 37°C, we have proposed that VP4, together with the N-terminal extensions of VP1, may form umbilical connections between PV and the membrane, which could serve to protect the genomic RNA from RNases during RNA transfer (22). In these asymmetric umbilicus-containing complexes, the width of the umbilicus is similar to the widths of filaments formed by VP4 peptides alone or by VP4 and viral RNA in heated virions (39) (Fig. S4). The length of the umbilical connection, which was fairly consistent from complex to complex and spanned the space between the virus surface and the membrane, could hypothetically accommodate two or three stacked VP4 pentamers. These structures, taken together with the information from the picornavirus literature that is listed above, provide a clear indication of the conformational repertoire of VP4 in its natural interactions with viral RNA. They suggest that VP4, either with or without other capsid proteins, might interact directly with the viral RNA to form a complicated RNA-protective structure during genome translocation.

10.1128/mBio.03695-20.4 FIG S4 VP4 forms stick structures with and without PV-RNA. (A) Electron micrograph showing that VP4 45-mer peptide contains a mixture of oligomers and fibrous aggregates. (B) Even in the absence of RNA, VP4-N45 forms stacked, stick-like structures at 37°C that can be visualized after staining with uranyl acetate by transmission electron microscopy. (C) Electron micrograph of polio-on-a-stick (POAS) structures. The poliovirus sample was heated at 50°C for 10 min and visualized after staining with uranyl acetate by electron microscopy. (D) POAS nucleoprotein fibers and attached virus particles remained intact, even after incubation for 1 h at 37°C in the presence of RNase A. (E) Electron micrograph of POAS nucleoprotein fibers and virus particles labeled with an antibody against VP4 protein. Gold-conjugated secondary antibodies are only evident where the virus is associated with a filamentous structure indicating that VP4 is one of the main components in POAS structures. (F) Electron micrograph showing stacked, stick-like structures in POAS fibers. Yellow arrows indicate the positions of stacked structures. Download FIG S4, JPG file, 0.6 MB. Copyright © 2021 Karunatilaka et al. 2021 Karunatilaka et al. https://creativecommons.org/licenses/by/4.0/ This content is distributed under the terms of the Creative Commons Attribution 4.0 International license.

Since the number of RNA nucleotides to be translocated during genome release vastly exceeds the limited VP4 supply, it requires either that RNA be threaded through a channel formed by VP4 and shift along the channel or that VP4 repeatedly dissociate from RNA at (or beyond) the membrane and then recycle, reattaching to newly emerging RNA close to the virus. The observed PV-RNA translocation time courses were indeed affected by the presence of exogenous VP4 45-mer (Fig. 3E and 4F), an observation consistent with the hypothesis that constantly shifting and changing VP4 interactions with RNA are necessary during viral RNA translocation. Further supporting our VP4 exchange model, mutant VP4-T28G significantly impacted the RNA translocation rates (Fig. 4I) by competing with full-length wild-type VP4 from the viral capsid, suggesting a dominant negative effect (and implying the existence of sequence-specific structures in the RNA-VP4 complex). Despite the finding that short linear protein-RNA complexes have previously been observed inside picornaviral empty capsids (32), our present results indicate that VP4 addition to umbilici must also take place outside the capsid. These novel findings, which favor a “VP4 recycling” mechanism, with significant on-rates and off-rates for VP4, provide a much more detailed picture of the infection process than previous studies permitted. Nevertheless, as discussed above, the absolute increase in fluorescence that was seen after VP4 addition (Fig. 5) and the incompleteness of RNA transfer (Fig. 5) might both be explained by VP4 release from the VP4-RNA complex being defective *in vitro*, in the absence of specific cellular factors or membrane components (which future experiments may identify). Future research is also needed to clarify the roles of both the N-terminal and C-terminal portions of VP4 and the effect of the T28G mutation on umbilicus formation and RNA translocation.

In summary, single-particle imaging of virus-bound receptor-decorated liposomes provides a useful cell-free system to investigate viral genome translocation. Based on our single-particle imaging studies, we propose that PV genome translocation is a complex, multistep process that involves an initial translocation burst with a series of RNA unwinding and propagation steps. Our real-time imaging results together with previous structural and genetic analysis support a model in which VP4 promotes RNA translocation by providing a protective channel during genome transfer. Besides the protection from RNases, interactions between the RNA genome and VP4 might also help maintain a single-stranded RNA structure for efficient translocation.

## MATERIALS AND METHODS

### Preparation of fluorophore-labeled poliovirus.

Poliovirus serotype I (Mahoney strain) was grown in suspension HeLa cells and purified as previously described (41). Briefly, infected cells were harvested by centrifugation, and virus was released by freeze-thawing the cell pellet. After removing the cell debris by low-speed centrifugation, virus was purified with a CsCl density gradient fractionation. Fluorophore-labeled poliovirus was obtained by labeling the PV capsid proteins with amine-reactive dye (Cy3-NHS ester; Molecular Probes). Purified virus was first incubated with Cy3 at room temperature for 30 min with gentle rocking. Then, the unbound dye was removed by buffer exchange using gel filtration columns (Nap5; GE Healthcare) as described elsewhere (20). The number of Cy3 dye molecules per virion (∼20 Cy3 dye molecules per PV) was estimated by measuring the absorbance at 260 nm and 550 nm to determine the concentration of poliovirus and Cy3 dye molecules in the purified, fluorophore-labeled virus solution, respectively. Virus titers and infectivity were determined using a standard plaque assay.

### Wild-type and mutant VP4 peptides.

N-terminally myristoylated wild-type (VP4-N45) and T28G mutant (VP4-T28G) VP4 N-terminal 45-mer peptides were synthesized with >95% purity by GenScript. To improve the solubility of both peptides, six lysines were added at the C terminus of the VP4 45-mer (31). The peptides were dissolved in sterile distilled water as recommended by the manufacturer.

### Infectivity assay.

Unlabeled and fluorophore-labeled PV titers were determined using plaque assays with adherent HeLa (S3) or Vero cells. Serial 10-fold dilutions of PV samples were added to cell monolayers in 6-well plates and incubated for 45 min at 37°C with rocking every 10 min. Growth medium containing 0.6% Noble agar (Sigma) was added to infected monolayers, and plates were kept for 30 min at room temperature. After incubation of plates for ∼48 h at 37°C, cells were stained with 0.2% crystal violet, and plaques were counted. Infectivity of fluorophore-labeled PV was expressed as a percentage of the control (unlabeled PV).

### Liposome preparation.

Liposomes were prepared by mixing phosphatidylethanolamine, phosphatidylcholine, sphingomyelin, cholesterol, and phosphatidic acid in chloroform (Avanti Polar Lipids) in a molar ratio of 1:1:1:1.5:0.3, respectively. Final concentrations of 8% (wt/wt) nickel salt of 1,2-dioleoyl-*sn*-glycero-3-[(*N*-(5-amino-1-carboxypentyl)iminodiacetic acid)succinyl] (DGS-NTA-Ni) and 1% (wt/wt) ammonium salt of 1,2-distearoyl-*sn*-glycero-3-phosphoethanolamine-*N*-[biotinyl(polyethylene glycol)-2000] (DSPE-PEG 2000 biotin) (Avanti Polar Lipids) were added to the lipid mix for binding of the His-tagged receptor to the liposomes and for surface immobilizing the RDLs for single-particle imaging experiments, respectively. Chloroform in the mixture was evaporated with a stream of argon to produce a thin lipid film, which was dried further under vacuum for >4 h. Dried lipid film was rehydrated to a final lipid concentration of 4 mg/ml using rehydration buffer (50 mM HEPES [pH 7.3], 50 mM NaCl and 1% glucose) containing 30 μM RNA binding dye TOTO-3 (Life Technology). TOTO-3 (excitation/emission wavelengths = 642/660 nm) was selected to monitor the viral RNA translocation for single-particle experiments with Cy3-labeled poliovirus, since it is a bright, far-red fluorescent dye that can be easily distinguished from Cy3 (excitation/emission wavelengths = 550/570 nm), and it is among the highest-sensitivity probes for nucleic acid detection. Liposomes were obtained by extruding the lipid mixture through a polycarbonate membrane with a 0.6- to 0.8-μm pore size (Avanti Polar Lipids).

### Formation of RDLs.

The soluble ectodomain (D1-3, without the cytoplasmic and transmembrane domains) of PVR with a six-histidine tag at the C terminus (sPVRHis) was a gift from the L. Shapiro lab (Department of Biochemistry and Molecular Biophysics, Columbia University, New York, NY). RDLs were made by incubating DGS-NTA-Ni-containing liposomes (∼4 mg/ml) with sPVRHis (0.5 mg/ml) at a 1:10 ratio (vol/vol) for 15 min at room temperature.

### Viral RNA extraction and binding of TOTO-3.

Viral RNA was extracted and purified from the poliovirus solution using a QIAamp viral RNA minikit (Qiagen) according to the manufacturer’s instructions (without adding carrier RNA). Fluorophore-labeled oligo(dT) containing biotin at the 5′ end [5′ biotin-(dT)_20_-Cy3 3′], which is complementary to the 3′ poly(A) tail of viral RNA, was used to immobilize the PV-RNA on a slide for imaging experiments. The PV-RNA–oligo(dT)-Cy3 complex was formed by heat-annealing a 2:1 mixture of PV-RNA and oligo(dT)-Cy3 at 85°C for 2 to 3 min and cooled for 15 to 20 min at room temperature. Preformed complex was added onto a streptavidin-coated slide, incubated for 15 min at room temperature, and washed with rehydration buffer to remove the unbound complexes. Excess TOTO-3 (30 μM) was added to the chamber and incubated in dark for 15 min at room temperature. Finally, the excess dye was removed by washing the slide with rehydration buffer containing an oxygen scavenger system (OSS; 1% glucose, 0.5 mg/ml glucose oxidase, and 34 μg/ml catalase), and the binding of TOTO-3 on full-length viral RNA was monitored by imaging the slide with both 561- and 642-nm lasers using a fluorescence microscope.

### Electron microscopy.

VP4 45-mer peptide was incubated for 15 to 30 min at 37°C in buffer containing 50 mM HEPES (pH 7.3) and 50 mM NaCl, adsorbed onto glow-discharged grids, and stained with 1% uranyl acetate to visualize the mixture of oligomers and fibrous aggregates. Polio-on-a-stick (POAS) structures were formed by heating the purified poliovirus sample at 50°C for 10 min and cooling on ice. POAS nucleoprotein fibers and virus particles were visualized by electron microscopy after adsorbing the diluted sample onto glow-discharged grids and stained with 1% uranyl acetate. The RNase-resistant nature of POAS fibers was determined after incubating the heated PV sample for 1 h at 37°C in the presence of RNase A, as previously described (39, 40). The grids were imaged at 80 kV in a JEOL 1200EX electron microscope equipped with an AMT 2k charge-coupled device (CCD) camera.

### Fluorescence microscopy.

To monitor the viral RNA translocation into the lumen of the TOTO-3 containing liposomes in bulk, a liposome-sPVRHis-PV (PV-RDL) complex was formed by mixing 4 μl of RDLs (4 mg/ml) and 2 μl of PV (0.5 mg/ml) in the presence of RNase A (50 μg/ml) in rehydration buffer. The reaction mixture (50 μl) was excited at 642 nm, and emission was recorded at 660 nm with a 5-nm slit width on a Varian Cary Eclipse fluorescence spectrophotometer under different reaction conditions, as mentioned above.

For single-particle imaging, TOTO-3-containing biotinylated RDLs (30 μl of 20 μg/ml) were added to a streptavidin-coated Ibidi μ-slide channel (Ibidi, Germany) and incubated for 15 min at room temperature to immobilize liposomes on a surface via a biotin-streptavidin linkage. After removing the unbound RDLs by washing with rehydration buffer, Cy3-labeled PV (36 ng) was added to the slide and incubated for 20 min at room temperature with rocking every 5 to 10 min. Unattached PV-Cy3 was removed by washing the channel with rehydration buffer containing an OSS (0.5 mg/ml glucose oxidase, 34 μg/ml catalase, and 1% glucose) to reduce photobleaching of fluorophores. Single-particle experiments with exogenous VP4 were performed by adding 1 μg of wild-type (VP4-N45) or mutant (VP4-T28G) VP4 N-terminal 45-mer peptide (GenScript) per channel for a final concentration of ∼3.4 μM. This represents an ∼650-fold molar excess relative to the total amount of VP4 in the virions added to the system prior to the washing step before peptide is added. However, this ignores the fact that the VP4 from virions is tethered and therefore present at very high local concentrations, whereas the exogenously added VP4 is freely diffusing. If we attempt to correct for the difference between constrained and freely diffusing contributions by assuming that all of the exogenously added VP4 within a sphere with twice the diameter of the average liposome is accessible to facilitate RNA translocation, this would correspond to 30× more added VP4 in the vicinity of a virus-liposome complex than that contributed by the individual virion in the complex. Note that both of these estimates are subject to assumptions that make their meaning questionable and that they are intended only to provide very rough estimates. Note also that because the added VP4 is a mix of fibers and smaller aggregates and it is not clear that all of the aggregates can participate in genome translocation, these are likely to represent overestimates of the fold excess.

Viral RNA translocation across the liposome membrane was monitored by time-lapse imaging of TOTO-3 encapsulated liposomes on a Nikon Ti motorized inverted microscope with a Perfect Focus System using a 1.49-numerical aperture (NA), 100× oil immersion Apo TIRF objective (Nikon Imaging Center, Harvard Medical School). The binding of PV-Cy3 on RDLs and binding of TOTO-3 on translocating RNA were monitored after exciting fluorophores with AOTF (acousto-optic tunable filter)-controlled 561-nm and 642-nm lasers (Spectral Applied Research), respectively. Fluorescence intensities were detected on a 512- by 512-pixel Hamamatsu ImagEM electron-multiplying charge-coupled device (EM-CCD) camera at 12 frames per min for 30 min with a 200-ms exposure time using appropriate filters (600/50 and 700/75; Chroma). All imaging experiments were performed at 37°C using a microscope incubation enclosure (Okolab).

### Image processing.

Single-particle movies were captured using Metamorph software, which saved individual frames as TIFF images. These TIFF images were imported into ImageJ and MATLAB for data analysis. First, movies were stabilized in ImageJ using the “align slices in stack” function. Next, the locations of the vesicles were identified from the red channel using a standard MATLAB spot finder tool. Aggregates of vesicles and vesicles that appeared to be smaller than two pixels in diameter were omitted from further analysis. The sums of the intensities of the pixels covering the area of each vesicle were calculated for each frame to generate a trace for each vesicle. These traces were imported into a custom MATLAB GUI, which used a hidden Markov model to determine when the viral genomic RNA translocated into the vesicle based on an increase in intensity in the red channel. The start and end times of the translocation were calculated by fitting the traces to a sigmoidal function (a logistic function in MATLAB). The start times for each trace were synchronized computationally, and the intensity values were binned as two-dimensional (2D) matrices. The matrices of intensity values were represented as colors to generate the synchronized heat maps. Translocation rates of individual virus particles were obtained from linear fitting the slope of the intensity trajectories. Histograms and their fits were prepared in MATLAB using standard built-in functions. Average translocation times and lag times were calculated by fitting the histograms with gamma distribution functions, and the error in the mean was obtained by bootstrapping.

The percentage of virus particles showing genome translocation was calculated by dividing the number of PV-RDL complexes showing an increase in fluorescence by the total number of PV-RDL complexes containing one virus per liposome under a given condition and multiplying the quotient by 100.
