# Supplementary figures and images for: Real-Time Imaging of Polioviral RNA Translocation across a Membrane
Source: mBio. 2021 Feb 23;12(1):e03695-20. doi: 10.1128/mBio.03695-20 (PMC8545138; doi:10.1128/mBio.03695-20)

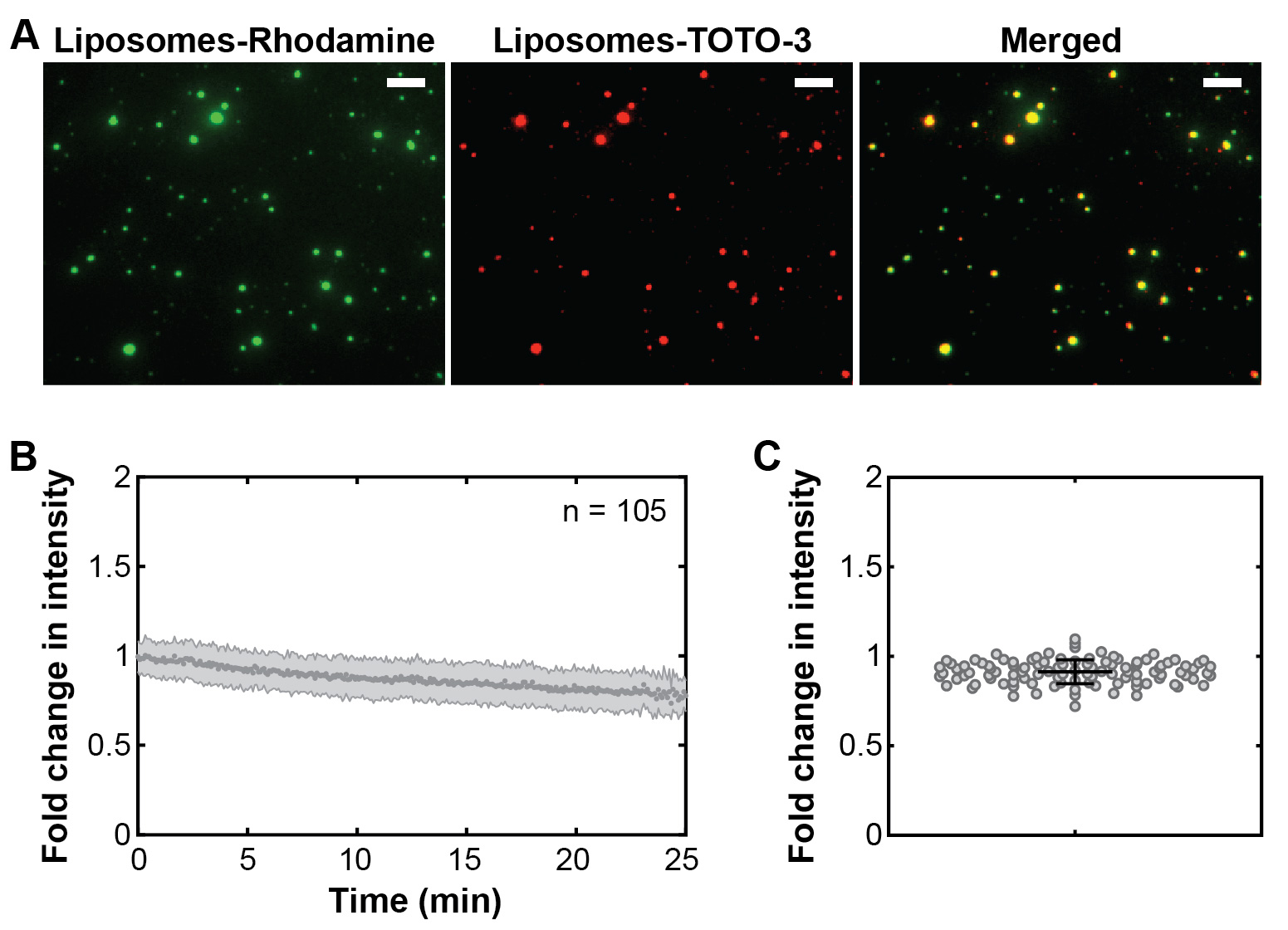

Supplement: FIG S1 [file mbio.03695-20-sf001.jpg]

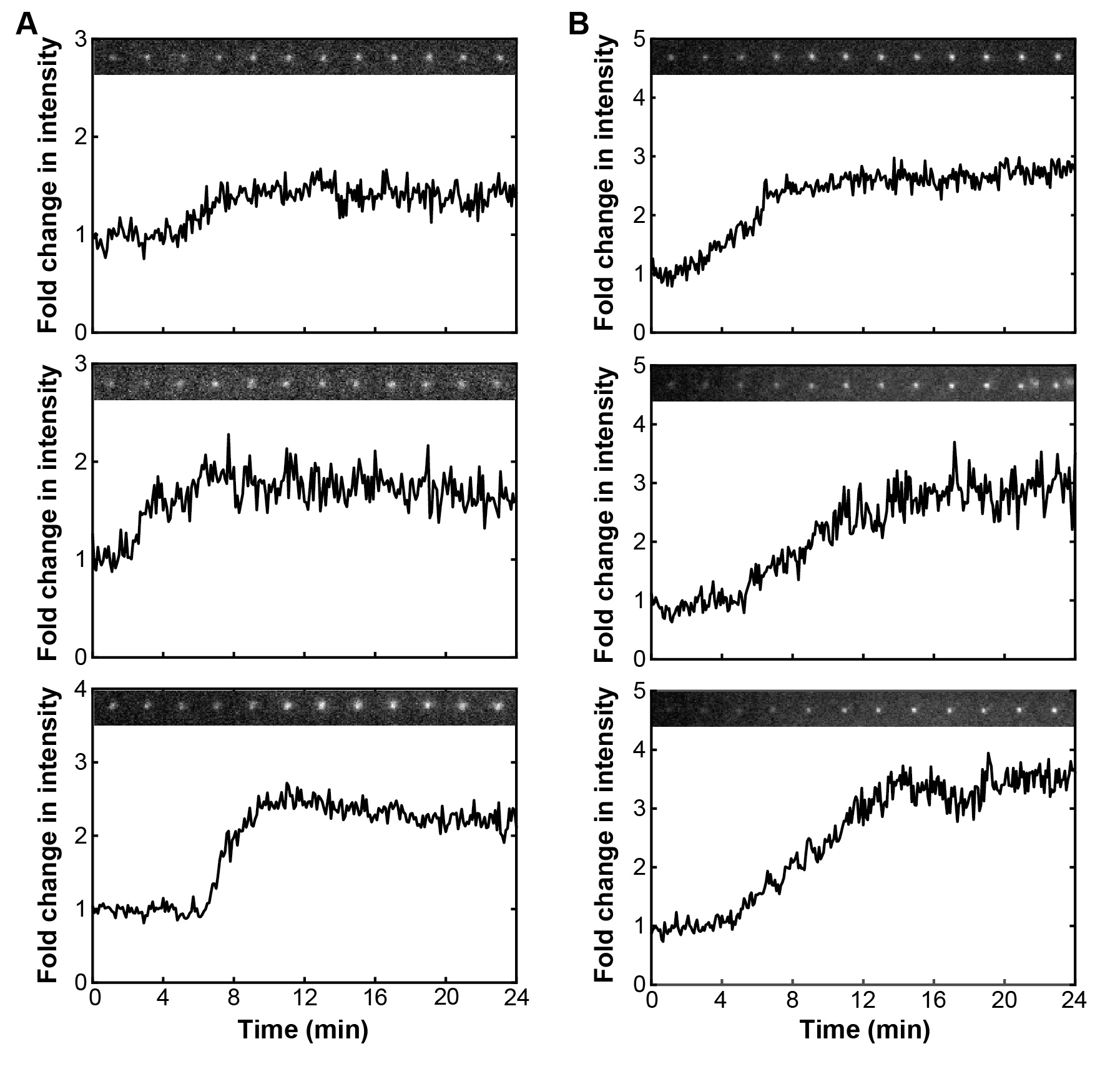

Supplement: FIG S2 [file mbio.03695-20-sf002.jpg]

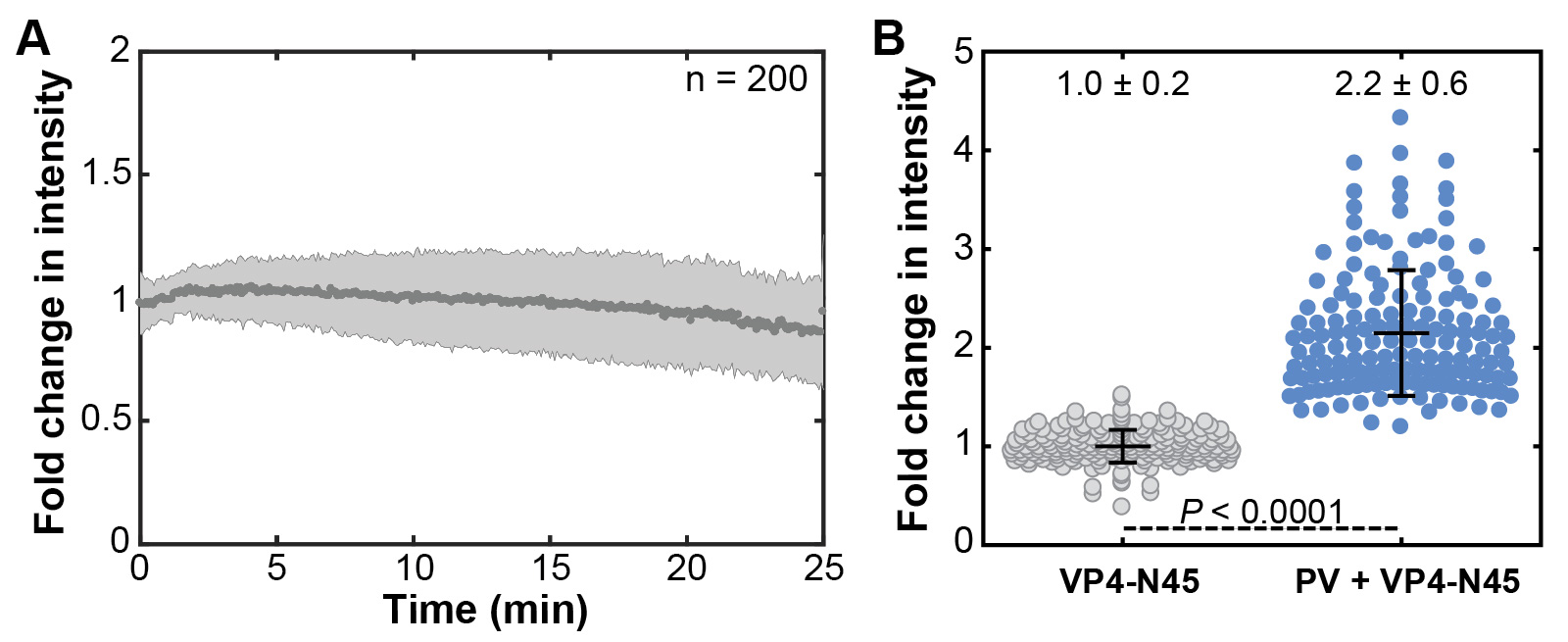

Supplement: FIG S3 [file mbio.03695-20-sf003.jpg]

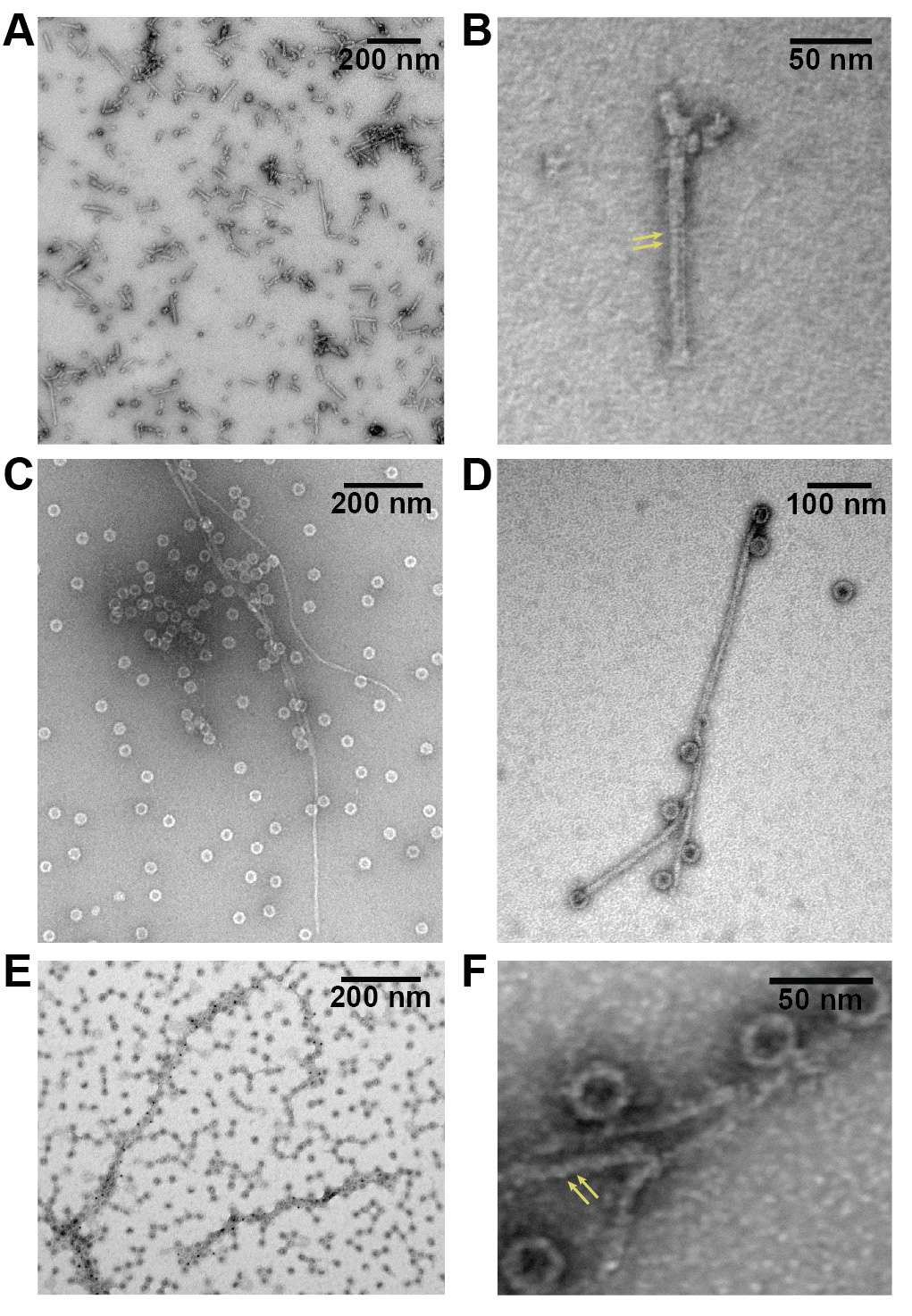

Supplement: FIG S4 [file mbio.03695-20-sf004.jpg]
